# Seventh International Symposium on Recent Advances in Environmental Health Research

**DOI:** 10.3390/ijerph8062565

**Published:** 2011-06-23

**Authors:** Paul B. Tchounwou

**Affiliations:** National Institutes of Health/National Center for Research Resources RCMI-Center for Environmental Health, College of Science, Engineering and Technology, Jackson State University, 1400 Lynch Street, Jackson, MS 39217, USA; E-Mail: paul.b.tchounwou@jsums.edu; Tel.: +1-601-979-0777; Fax: +1-601-979-0570

This special issue of *International Journal of Environmental Research and Public Health* is dedicated to the publication of selected papers presented at the *Seventh International Symposium on Recent Advances in Environmental Health Research.* The Symposium was organized by Jackson State University (JSU) from September 12–15, 2010 at the Marriott Hotel in Jackson, Mississippi. It was built upon the overwhelming success of previous symposia hosted by JSU and co-sponsored by the National Institutes of Health (NIH) RCMI-Center for Environmental Health, the U.S. Department of Education Title III Graduate Education Program, the U.S. Environmental Protection Agency, the JSU Office of Academic Affairs, and the JSU Office of Research and Federal Relations.

In sustaining the tradition initiated with the first international symposium in 2004, the program of the *Seventh International Symposium on Recent Advances in Environmental Health Research* provided a strong forum to discover the latest scientific advances in our search for global solutions to major environmental and human health challenges. The Symposium’s program had continued to have a special appeal to national and international scientists who have been committed to bioenvironmental and public health research; studying the toxic mechanisms of action of various environmental agents, developing new approaches for detecting or remedying environmental damage, identifying and characterizing genes involved in the manifestation of environmentally-related diseases, conducting basic and translational research, and providing the public and policy makers with scientific tools that are critical for environmental and human health decision-making [1]. This comprehensive approach offers practical and cost-effective strategies to improving environmental quality and public health though translation of new scientific knowledge and discoveries from fundamental and basic sciences to environmental sustainability and public health protection. Hence, the symposium was of special interest to toxicologists, environmental chemists and biologists, epidemiologists, public health officials, and civil and environmental engineers having an interest in environmental and public health research. Also, the symposium series continued to offer unparalleled opportunities for networking and exchange of ideas, leading to scientific collaborations, resources sharing, and strategic planning for multi- and inter-disciplinary approaches to environmental and public health research.

Building on the foundation of the first (2004), second (2005), third (2006), fourth (2007), fifth (2008) and sixth (2009) symposia, the *Seventh International Symposium on Recent Advances in Environmental Health Research (2010)* served as a platform to exchange innovative ideas and communicate the latest advances in scientific research and new developments on important environmental and human health topics, including the following:

- Nanoscience, Nanotechnology and Nanotoxicology
- New Frontiers in Environmental Health Research
- Environmental Toxicology and Health Risk Assessment
- Emerging Topics in Computational Biology, and Environmental Modeling
- Health Disparities and Environmental Security
- Medical Geology and Human Health
- Natural Resources Damage Assessment and Management

Detailed information on each of these topics has been previously published [1].

The symposium attracted 298 participants from 21 countries representing all five continents. A total of 199 scientific presentations were made across the disciplines of environmental health, biomedical and clinical sciences, and public health. As listed above, the scientific program was composed of seven plenary sessions where oral/plenary presentations were given by forty two invited speakers. In addition, there were two poster sessions—one for faculty and professional scientists, and one for students that included awards for best posters presentations at four levels of the educational pipeline including high school, undergraduate, master’s and doctorate levels. Three certificates and prizes (first, second and third) were awarded for each education level.

Original contributions were solicited on relevant areas of interest to environmental research and public health. Authors were asked to access the journal’s website and submit their full length manuscripts. These manuscripts were processed and sent out for peer-review by environmental and public health experts in their respective fields. A rigorous peer-review process was conducted as previously described [1], and according to the high publication standard of International Journal of Environmental Research and Public Health [2].

I wish to extend special thanks to Dr. Richard E. Price (Retired Chief of the Division of Environmental Processes and Engineering, Environmental Laboratory at U.S. Army Engineer Research and Development Center-ERDC) for serving as Distinguished Speaker for the Biomedical Sciences and Health Information Lecture Series that is held in conjunction with the Symposium. Dr. Price presented the mission and strategic goals of ERDC; a unique organization designed to conduct highly focused research and to develop technologies that support the U.S. Corps of Engineers, the Department of Army, and the Nation. He also provided valuable information on specialized ERDC research and training in the areas of contaminants assessment and remediation. Other plenary presentations and keynote addresses were made by prominent biomedical and environmental health scientists with research expertise in cancer, diabetes, HIV/AIDS, infectious and parasitic diseases, cardiovascular diseases, neurodegenerative diseases, gene-environment interactions, nanoscience and nanomedicine, emerging technologies, health disparities and other environmentally-related illnesses. These important health issues were associated with the symposium topics.

Session chairpersons included Dr. Farshad Amini, Jackson State University, School of Engineering, Jackson, Mississippi, USA; Dr. Stella Anyangwe, World Health Organization Country Office, Pretoria, South Africa; Dr. Mario Azevedo, Jackson State University, College of Public Service, Jackson, Mississippi, USA; Dr. Jose A. Centeno, Armed Forces Institute of Pathology, Washington DC, USA; Dr. Edmond Creppy, University of Bordeaux, Faculty of Pharmacy, Bordeaux, France; Dr. Waneene Dorsey, Grambling State University School of Arts and Science, Grambling, Louisiana, USA; Dr. Andrew J. Englande, Tulane University, School of Public Health & Tropical Medicine, New Orleans, Louisiana, USA; Dr. Ramzi Kafoury, Jackson State University, School of Science and Technology, Jackson, Mississippi, USA; Dr. Joseph Landolph, University of Southern California, School of Pharmacy, Los Angeles, California, USA; Dr. Danuta Leszczynska, Jackson State University, School of Engineering, Jackson, Mississippi, USA; Dr. James Maddirala, Jackson State University, Jackson, Mississippi, USA; Dr. Nelly Manay, University of the Republic, Faculty of Chemistry, Montevideo, Uruguay; Dr. Dora N. Mbanya, University of Yaounde, Faculty of Medicine, Yaounde, Cameroon; Dr. Loretta Moore, Jackson State University, School of Engineering, Jackson, Mississippi, USA; Dr. Marinelle Payton, Jackson State University, School of Health Science, Jackson, Mississippi, USA; Dr. Hector Rubio-Arias, Autonomous University of Chihuahua, College of Science, Chihuahua, Mexico; Dr. Karam Soliman, Florida A&M University, College of Pharmacy, Tallahassee, Florida, USA; Dr. William M. Southerland, Howard University, College of Medicine, Washington DC, USA; Dr. Wilbur Walters, Jr., Jackson State University, School of Science and Technology, Jackson, Mississippi, USA; and Dr. Yongtao Yu, Jackson State University, School of Science and Technology, Jackson, Mississippi, USA.

Special thanks are extended to Mrs. Rose Foster and Mrs. Wilma Templin-Branner of Oak Ridge Institute for Science and Education, Dr. Kenneth Ndebele and Dr. Barbara Graham for their continued support and help with the organization of the pre-symposium workshop on the *National Library of Medicine Web-Based Resources for Environmental Health and Biomedical Research.* Major emphasis of the workshop was training participants on how to access and retrieve important environmental health and biomedical research information from the Toxicology Network database and other relevant web-based biomedical resources.

Special thanks are also extended Dr. Leslie B. McLemore (Interim President), Dr. Quinton L. Williams (Interim Provost & Vice President for Academic Affairs), Dr. Felix Okojie (Vice President for Research and Federal Relations), Dr. Mark Hardy (CSET Dean) and Dr. Mary Myles (Director of Title III Program) for their administrative support.

I would like to commend the authors for their involvement and cooperation, and for their outstanding contributions to advancing scientific research and facilitating informed decision making in the critical area of environmental sustainability and public health protection. Special thanks are also extended to all the peer-reviewers who took time from their busy schedules to carefully and critically review each of the manuscripts. Special appreciations are also extended to all my colleagues and staff who worked very hard to make the symposium a total success.
